# Severe Occupational Hypersensitivity Pneumonitis: A Case Series of Four Patients Requiring Lung Transplantation

**DOI:** 10.1002/ajim.70070

**Published:** 2026-03-17

**Authors:** Ludwig Frei‐Stuber, Judith Mohren, Ester Mau, Bernhard Werner, Rudolf A. Hatz, Jürgen Barton, Dennis Nowak

**Affiliations:** ^1^ Institute and Clinic for Occupational, Social and Environmental Medicine, Comprehensive Allergy Center (CAC) University Hospital, Ludwig‐Maximilians‐Universität (LMU) Munich Munich Bavaria Germany; ^2^ German Center for Lung Research (Deutsches Zentrum für Lungenforschung e. V., DZL), Comprehensive Pneumology Center Munich (CPC‐M) University Hospital, Ludwig‐Maximilians‐Universität (LMU) Munich Munich Bavaria Germany; ^3^ Department of Thoracic Surgery, Comprehensive Cancer Center (CCC) University Hospital, Ludwig‐Maximilians‐Universität (LMU) Munich Munich Bavaria Germany; ^4^ Department of Medicine V (Pneumology), Comprehensive Allergy Center (CAC) University Hospital, Ludwig‐Maximilians‐Universität (LMU) Munich Munich Bavaria Germany

**Keywords:** antigen avoidance, early recognition, extrinsic allergic alveolitis, lung transplantation, occupational diseases, occupational exposure, occupational hypersensitivity pneumonitis, preventive occupational medicine, respiratory insufficiency, work‐related interstitial lung disease

## Abstract

Hypersensitivity pneumonitis (HP) is an immune‐mediated interstitial lung disease triggered by repeated inhalation of organic or chemical antigens. Occupational exposures account for approximately 19% of all cases. Early diagnosis, identification of the responsible antigen(s), and immediate avoidance of exposure are crucial to prevent irreversible pulmonary fibrosis. However, HP often remains unrecognized or is misclassified as another respiratory disorder such as asthma, chronic obstructive pulmonary disease (COPD), or idiopathic pulmonary fibrosis. As a result, the causal link between symptoms and workplace exposure is frequently established only in advanced disease stages—or not at all. Such delays may result in chronic respiratory failure, occupational disability, prolonged oxygen therapy, and, in severe cases, lung transplantation. We report four patients in whom HP was ultimately recognized as an occupational disease or recommended for legal recognition in court. At the time of diagnosis, all cases had progressed to advanced, fibrotic HP, rendering both primary and secondary prevention impossible. In each instance, earlier identification of the occupational trigger followed by immediate antigen avoidance could likely have prevented the development of irreversible lung damage. This case series underscores the need for early and comprehensive pulmonary assessment, including detailed occupational history‐taking, serologic and radiologic evaluation, and prompt referral to an occupational physician when HP is suspected. Close interdisciplinary collaboration between pulmonologists and occupational medicine specialists is essential to reduce diagnostic latency, prevent progression to end‐stage lung disease, and improve clinical and socioeconomic outcomes.

## 1 Introduction

Hypersensitivity pneumonitis (HP), also known as extrinsic allergic alveolitis (EAA), is an interstitial lung disease caused by an exaggerated and dysregulated immune response to inhaled antigens. The disease typically develops after sensitization from repeated exposure to aerosolized organic or chemical agents resulting [1].

More than 200 antigens have been identified as potential triggers of HP. Exposure may occur in occupational environments, at home, or during leisure activities. Most causative agents are derived from fungi, bacteria, protozoa, animal proteins, or low‐molecular‐weight chemical compounds [2]. Specific terms have been established for HP subtypes according to the causal antigen. Examples include farmer's lung (moldy hay), humidifier lung (contaminated water), baker's lung (Aspergillus‐derived enzymes in baking agents), bird fancier's or bird breeder's disease (avian droppings, serum, and feathers), fish feed or fish meal alveolitis, isocyanate alveolitis, painter's lung (triglycidyl isocyanurate), and drug‐induced HP (penicillins, cephalosporins, methotrexate, α‐interferon, lenalidomide, pravastatin, temozolomide) [3]. In 20%–60% of patients with HP, the causative antigen cannot be identified [4, 5].

The prevalence of HP varies regionally, depending on climatic, occupational, and environmental factors. U.S. insurance data report an estimated annual prevalence of 1.67–2.71 cases per 100,000 individuals [3, 6]. The true prevalence is likely higher due to frequent misdiagnosis and underreporting [7, 8]. Among interstitial lung diseases, HP accounts for approximately 10% of cases [9, 10]. Chronic fibrotic HP carries a poor prognosis, often with progressive decline in general health. Even with antifibrotic and immunosuppressive therapy, 5‐year survival remains around 50%, and many patients ultimately require lung transplantation [7, 11, 12]. Approximately 19% of HP cases are attributable to occupational exposures [13, 14].

Diagnostic criteria for acute and chronic occupational HP, based on the review by Quirce et al. (2016) [15] and the recently published S2k guideline on HP by Koschel et al. (2024) [16] are summarized in Table 1. As with other lung diseases, early detection remains a critical prognostic factor [17, 18, 19, 20].

**Table 1:** Diagnostic criteria for acute (non‐fibrotic) and chronic (fibrotic) hypersensitivity pneumonitis (HP).^1^

Diagnostic category	Criterion	Source and notes
Acute/non‐fibrotic HP	All five of the following criteria must be met:	—
	1. Potential exposure to antigens	Both Quirce (2016) and Koschel (2024)
	2. Recurrent symptoms 4–8 h after exposure	Both
	3. Elevated specific IgG antibodies against a potential antigen	Both; emphasized by Koschel (2024) as exposure marker only
	4. Inspiratory crackles on lung auscultation	Described by Quirce (2016)
	5. HRCT findings consistent with inflammatory HP	Both; defined in more detail by Koschel (2024)
	If one or more criteria are not fulfilled, they may be replaced by one of the following invasive criteria:	—
	6. Lymphocytosis ( > 30%) in bronchoalveolar lavage (BAL)	Both; threshold defined by Koschel (2024)
	7. Histopathologic findings typical or likely of acute/non‐fibrotic HP	Both
	8. Positive inhalation challenge, re‐exposure, or abstinence test	Described by Quirce (2016) only
Chronic/fibrotic HP	Diagnosis requires ≥ 3 of the following criteria:	—
	1. Potential exposure to antigens	Both
	2. Elevated specific IgG antibodies and/or BAL lymphocytosis ( > 30%)	Both; numeric threshold from Koschel (2024)
	3. HRCT findings consistent with fibrotic HP	Both
	4. Histopathologic findings typical or likely of chronic fibrotic HP	Both; terminology harmonized by Koschel (2024)

1 (According to Quirce et al., 2016 and Koschel et al., 2024)

Abbreviations: BAL, bronchoalveolar lavage; HP, hypersensitivity pneumonitis; HRCT, high‐resolution computed tomography.

Here, we present a case series of four patients with occupational HP in whom delayed diagnosis led to severe, progressive disease requiring lung transplantation. Earlier recognition could likely have prevented these outcomes.

The following search criteria were applied to a review of the complete database of all patients consulting our outpatient clinic, including, where necessary, a review of available medical records and expert reports:

- Reason for consultation: medical consultations covered by statutory health insurance, pre‐employment medical examinations, other fitness‐for‐duty assessments (e.g., for driving, supervisory, or monitoring tasks), preventive medical examinations, and expert reports for statutory accident insurance institutions and courts
- Type of examination request: in‐person evaluation at our outpatient clinic or assessment based on a file review
- Time period: January 1, 2023, to December 31, 2024
- Presence of HP as the primary diagnosis
- Etiology of HP (occupational or non‐occupational, including identification of the causative antigen where possible)

- Treatment of HP

The inclusion criteria for cases were as follows:

- Preparation of expert reports for statutory accident insurance institutions or courts
- In‐person evaluation at our outpatient clinic
- Time period: January 1, 2023, to December 31, 2024
- Primary diagnosis of HP
- Occupational causation of HP, preferably with identification of the causative antigen and recognition by the responsible statutory accident insurance institution (in Germany, HP corresponds to Occupational Disease No. 4201 according to the Occupational Diseases Ordinance)
- Bilateral lung transplantation due to occupational HP

All inclusion criteria had to be fulfilled. Consequently, no additional patients meeting all criteria were identified during the study period, resulting in a consecutive case series. Among all patients diagnosed with HP during the same time frame, these four patients accounted for 11.1% of cases.

All patients provided written informed consent for anonymous publication of their medical data. Ethics approval was not required for this retrospective case series.

Language editing assistance was provided by ChatGPT (OpenAI, San Francisco, CA, USA) under the supervision of the corresponding author.

## 2 Case Report 1

### 2.1 Occupational History

After graduating from secondary school in 1990, Patient 1 (female, 46 years at initial diagnosis, 53 years at occupational assessment in our clinic; height 168 cm, weight 74 kg) completed a 3‐year apprenticeship as a seamstress (1990–1993). She subsequently worked for 6 years (1993–1999) inspecting circuit boards and for 8 years (1999–2007) in cable assembly. Thereafter, she was employed as a seamstress in a balloon factory for 4 years (2007–2011). During the following 9 years (2011–2020), she worked in a large gardening business. This employer also operated a department dedicated to the breeding and sale of birds, in which Patient 1 was regularly employed, resulting in occupational exposure to avian antigens. Due to progressive respiratory symptoms, she was forced to leave employment in 2020. At that time, there was a high risk of complete work incapacity and premature retirement; however, these outcomes were fortunately averted in subsequent years due to successful bilateral lung transplantation and the cooperation of the employer.

### 2.2 Clinical Course Until Bilateral Lung Transplantation

Cough and dyspnea first developed in 2012. A connection between symptoms and occupational exposure was not initially recognized. Prior to the final diagnosis, chronic obstructive bronchitis and asthma had been considered. The diagnosis of HP was established only after a 4‐year delay (in 2016), despite multiple consultations at a pulmonology practice, and was ultimately made during evaluation by another physician at a specialized pulmonary clinic. At that time, chronic obstructive pulmonary disease (COPD; Global Initiative for Chronic Obstructive Lung Disease (GOLD) stage IV) was also diagnosed, considered most likely secondary to HP. Cumulative tobacco consumption was less than one pack‐year and had ceased more than 20 years earlier; α‐1‐antitrypsin deficiency was excluded.

Computed tomography (CT) of the chest revealed ground‐glass opacities and predominantly cicatricial, emphysematous, and interstitial parenchymal changes with pronounced bullous transformation in 2015, which had further progressed after 4 years (2019, Figure 1). Bronchoalveolar lavage demonstrated lymphocytic alveolitis. Due to the late diagnosis and delayed recognition of the occupational association, respiratory insufficiency had developed about 7 years earlier (2012), necessitating long‐term oxygen therapy from 2015 onward. In 2017, moderate pre‐ and post‐capillary pulmonary hypertension was diagnosed, while coronary artery disease was invasively excluded. Bilateral lung transplantation was eventually performed in 2020 because of severe restrictive impairment and markedly reduced diffusion capacity. Still in 2020, as the patient's pulmonary findings worsened despite ongoing work and given that occupational antigens are frequently implicated in HP, the university thoracic surgery center subsequently raised suspicion of an occupational disease.

**Figure 1:**
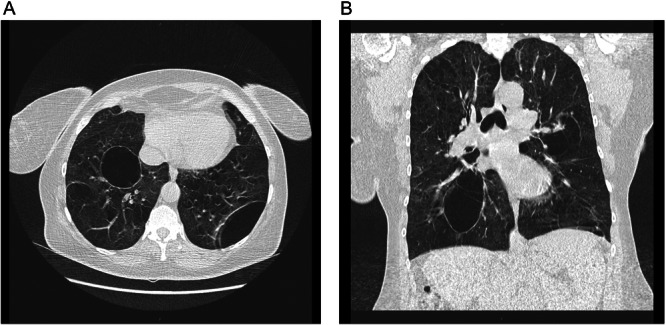
Chest computed tomography (CT) of Patient 1 (2019) in axial (A, mid‐to‐basal) and coronal (B) planes, lung window. Findings: Ground‐glass opacities with predominantly cicatricial, emphysematous, and interstitial fibrotic changes, as well as pronounced bullous alterations.

### 2.3 Clinical Course After Bilateral Lung Transplantation

Following transplantation, numerous complications occurred, including pulmonary embolism requiring parenteral anticoagulation, which was complicated by bleeding from a Forrest III gastric ulcer and diverticular hemorrhage. Recurrent infections, including atypical pneumonia, were documented. Pleural effusions required repeated thoracenteses. Osteoporosis developed under steroid therapy. During immunosuppressive therapy, pancytopenia and recurrent cytomegalovirus (CMV) reactivation were noted, necessitating treatment with ganciclovir and anti‐CMV immunoglobulins. Episodes of acute kidney injury occurred repeatedly, eventually leading to chronic renal impairment.

In 2021, obstructive sleep apnea syndrome was diagnosed (body mass index ≈ 26.2 kg/m²) and treated with nocturnal biphasic positive airway pressure (BiPAP) ventilation. Arterial hypertension was preexisting.

### 2.4 Occupational Medical Assessment

At the occupational assessment in our outpatient clinic in 2023, the primary focus was determining the degree of work‐related disability. HP with bilateral lung transplantation represents near‐maximal organ damage attributable to this disease.

During exertion (6‐min walk test), arterial pO₂ decreased pathologically from 65.7 to 58.5 mmHg. Despite approximately 5 years of antigen avoidance, specific IgG antibodies against duck, canary, and budgerigar feathers, serum, and feces, as well as pigeon antigens, remained moderately elevated. The patient reported that canaries, budgerigars, and pigeons were kept and sold in a section of the gardening center, where she occasionally assisted with animal care. The elevated anti‐duck‐feather IgG was likely due to cross‐reactivity, as no relevant occupational or private exposure to ducks was known.

Symptoms were absent at rest, but marked exertional dyspnea persisted. The patient could ascend only one flight of stairs slowly and required rest afterwards. Long‐term oxygen therapy had been necessary for 5 years before transplantation and discontinued a few weeks afterwards (2015–2020). Cognitive endurance was limited to a few hours per day. Although diffusion capacity had improved compared with pre‐transplant levels (27% predicted; 34.5% of lower limit of normal [LLN]), it remained moderately reduced (56% predicted; 72.1% of LLN).

From our perspective, no professional activities were possible from the time of listing for transplantation (severe restrictive defect, reduced diffusion capacity, long‐term oxygen therapy) until oxygen therapy cessation following transplantation. Thereafter, limited part‐time light physical activity was deemed feasible—ideally within a reintegration program, avoiding strenuous work and antigen exposure. Pulmonary rehabilitation and re‐evaluation after 2 years were recommended.

Through internal job restructuring, she was reassigned to selling finished furniture outside the gardening area, enabling successful occupational reintegration.

## 3 Case Report 2

### 3.1 Occupational History

After graduating from secondary school in 1977, Patient 2 (male, 56 years at initial diagnosis, 63 years at occupational assessment in our clinic; height 190 cm, weight 106 kg) completed a 3‐year apprenticeship as a painter (1977‐1980), followed by 18 months of military service (1980–1981). He subsequently worked as a zoo caretaker for approximately 10 years (1981–1991), performing various manual tasks without direct animal contact for about 5 additional years (1991–1996). For the next 20 years (1996–2016), he was employed by the municipality as a maintenance worker and painter.

A basement room in the administration building served as a staff lounge for breaks and overnight on‐call duties. Upon his assuming this position, the area already had a musty, moldy odor. Two years later, replacement of the wooden wall paneling revealed extensive mold growth. According to the patient, the mold was repeatedly painted over but recurred continuously. After approximately 20 years of exposure in this basement environment, he was forced to stop working due to progressive respiratory distress.

### 3.2 Clinical Course Until Bilateral Lung Transplantation

Soon after starting this job (1997), the patient developed cough and dyspnea, particularly during respiratory infections accompanied by fever lasting about one week. His general practitioner referred him to a cardiologist, who found no cardiac explanation. Seven years later (2004), he experienced syncope, prompting further cardiologic and pulmonologic evaluation—again without identification of a cause.

In 2010, worsening cough and dyspnea prompted consultation with another pulmonologist, who ordered chest CT and lung biopsy, leading to histological diagnosis of interstitial pulmonary fibrosis. At that time, the patient was diagnosed with idiopathic pulmonary fibrosis. Progressive respiratory insufficiency required continuous nocturnal and intermittent daytime oxygen supplementation.

After an additional 6 years, the patient was referred to a university interstitial lung disease clinic, where terminal‐stage HP was diagnosed in 2016. CT imaging demonstrated pulmonary fibrosis and emphysema without suspicious nodules and with cardiomegaly (2016). Serum testing revealed elevated specific IgG antibodies against mold antigens, confirming the occupational trigger. The patient was listed for transplantation, and bilateral lung transplantation was performed a few months later (still in 2016).

### 3.3 Clinical Course After Bilateral Lung Transplantation

Post‐transplant immunosuppressive therapy led to chronic renal insufficiency, diabetes mellitus, and glucocorticoid‐induced osteoporosis. Bilateral cataracts developed several months later and were successfully treated surgically. A spontaneous thoracic vertebral fracture occurred and was managed conservatively.

In 2022, the patient contracted severe COVID‐19 (unvaccinated at that time) and was treated with nirmatrelvir (600 mg/day), ritonavir (200 mg/day), and intravenous SARS‐CoV‐2 immunoglobulins. Despite treatment, he developed pneumonia and bilateral upper‐limb paresis, which only partially resolved. Short‐term memory deterioration has persisted since then.

The patient himself raised the suspicion of an occupational disease in 2016, as his treating pulmonologists had not explored workplace factors. During a legal dispute over compensation, samples were obtained from the renovated basement in question. Microbiologic testing confirmed persistent significant mold contamination. The patient denied any relevant private mold exposure.

### 3.4 Occupational Medical Assessment

At the occupational medical evaluation in our clinic in 2023, the key question concerned early retirement eligibility and potential for reemployment in another capacity.

Diagnostic findings are summarized in Table 2. Spirometry and body plethysmography revealed no obstructive ventilatory defect; total lung capacity (TLC) was 80% predicted (100.0% of LLN), consistent with a borderline restrictive pattern. Forced vital capacity (FVC) measured 3.92 L—slightly above the LLN according to the Global Lung Initiative (GLI, Quanjer et al., 2012 [21]) of 3.84 L. The 6‐min walk distance was 38.7% of the age‐ and weight‐adjusted target, though not objectively confirmed due to severe asthenia precluding cardiopulmonary exercise testing. Diffusion capacity improved markedly compared with pre‐transplant levels (17% predicted; 22.5% of LLN) but remained mildly reduced (67% predicted; 89.9% of LLN). Specific IgG levels against ubiquitous mold antigens were slightly elevated.

**Table 2:** Clinical and occupational overview of the four patients with hypersensitivity pneumonitis (HP).

Parameter	Patient 1	Patient 2	Patient 3	Patient 4
Gender	Female	Male	Male	Male
Time of initial diagnosis (year)	2016	2016	2020	2008
Age at initial diagnosis (years)	46	56	57	40
Time of occupational assessment (year)	2023	2023	2024	2023
Age at occupational assessment (years)	53	63	61	55
Occupation	Employee in gardening center	Domestic craftsman/painter	Farmer	Seed dealer (minor farming)
Antigen source	Avian antigens (duck, canary, budgerigar, pigeon)	Mold (workplace basement)	Agricultural/barley dust	Mold (seed contamination)
Tobacco use	< 1 pack‐year, abstinent > 20 years	Never‐smoker	Never‐smoker	Never‐smoker
Latency: symptoms → diagnosis	4 years	19 years	11 years	10 years
Latency: diagnosis → report of suspected occupational disease	4 years	1 month	4 months	1 month
Reporter of suspected disease	University thoracic surgery center	Patient (self‐report)	Treating pulmonologist	Treating pulmonologist
Oxygen therapy before transplantation	Yes	Yes	Yes	Yes
Time of bilateral lung transplantation (year)	2020	2016	2023	2024
Oxygen therapy after transplantation	No (discontinued within weeks)	No	No	No
Pulmonary function before transplantation	Severe obstruction/restriction (FEV₁ 21%, FVC 51%, TLC 139%)	Severe restriction (FEV₁ 32%, FVC 54%, TLC 56%)	Moderate restriction (FEV₁ 68%, FVC 47%, TLC 47%)	Severe restriction (FEV₁ 49%, FVC 55%, TLC 40%)
TLC before transplantation (% of LLN)	169.9	69.5	58.6	49.6
Diffusion capacity before transplantation (% predicted)	27	17	39	37
Diffusion capacity before transplantation (% of LLN)	34.5	22.5	52.4	48.9
Pulmonary function after transplantation	Improved (FEV₁ 70%, FVC 71%, TLC 62%)	Improved (FEV₁ 74%, FVC 77%, TLC 80%)	Improved (FEV₁ 80%, FVC 67%, TLC 69%)	Improved (FEV₁ 81%, FVC 73%, TLC 72%)
TLC after transplantation (% of LLN)	76.0	100.0	86.1	89.5
Diffusion capacity after transplantation (% predicted)	56	67	82	64
Diffusion capacity after transplantation (% of LLN)	72.1	89.9	109.6	84.8
Specific IgG	Persistently elevated to avian antigens	Slightly elevated mold‐specific IgG	Negative (thermophilic actinomycetes, fungi and other organisms including Candida albicans and barley)	Elevated mold‐specific IgG
CT findings (pre‐transplant)	Ground‐glass opacities, cicatricial emphysema, bullous changes	Fibrosis, emphysema, cardiomegaly	Reticulations without honeycombing	Ground‐glass infiltrates, micronodules, fibrosis, cardiomegaly
Histopathology of explanted lungs	Granulomatous bronchitis and bronchiolitis obliterans, no malignancy	Lymphocytic interstitial infiltrates, granulomas, no malignancy	Organizing pneumonia with granulomas, no malignancy	Broad interstitial fibrosis, fibroblast proliferation, no malignancy
Return to work after transplantation	Yes (reassigned to sales position)	Yes (administrative duties)	Yes (light agricultural work with assistance)	Pending (reassessment ongoing)

Abbreviations: BAL, bronchoalveolar lavage; DLCO, diffusing capacity for carbon monoxide; HP, hypersensitivity pneumonitis; FEV₁, forced expiratory volume in one second; FVC, forced vital capacity; HRCT, high‐resolution computed tomography; LLN, lower limit of normal; TLC, total lung capacity.

Following COVID‐19, paresis of the right arm, distal paresthesia, cognitive deficits, and fatigue persisted. The patient could ascend stairs slowly and only with pauses. Long‐term oxygen therapy had been required for 6 years prior to transplantation but was discontinued afterward.

The prolonged disease course and compensation litigation contributed to depressive symptoms managed with psychotherapy. A physically light, full‐time position without shift work was deemed feasible. The recommendations were implemented successfully, and the patient was reassigned to administrative duties in the municipal archives. Because neurological deficits remained predominant, neurorehabilitation was advised, along with optimized, guideline‐based treatment of the depressive disorder.

## 4 Case Report 3

### 4.1 Occupational History

Patient 3 (male, 57 years at initial diagnosis, 61 years at occupational assessment in our clinic, height 180 cm, weight 76 kg) completed secondary school in 1981 and then began a 3‐year (1981–1984) apprenticeship as a farmer on his parents' farm, where he subsequently worked full time for an additional 28 years (1984–2012). He later worked for approximately 10 years (2012–2022) as a machinist and crane operator.

Growing up in an agricultural environment, the patient regularly assisted on the family farm, where cows and pigs were kept, and various crops were cultivated. Pig farming was discontinued first, whereas dairy farming continued until approximately 15 years ago (2010). The patient reported no clear symptoms of bronchial asthma or acute HP when working in the stables; however, he experienced annual febrile episodes lasting about 2 weeks during barley harvests. The stables were rebuilt about 25 years ago (2000), and since then, barley straw was no longer used as bedding except for small quantities for calf breeding.

Barley cultivation continued, although harvesting was later outsourced to external contractors. Straw was stored in the hayloft using a blower and distributed manually. The patient reported long‐standing exertional dyspnea during agricultural work since 2009 but could not specify which activities triggered his symptoms.

### 4.2 Clinical Course Until Bilateral Lung Transplantation

Symptoms including cough, dyspnea, and fever associated with barley exposure persisted, but the patient continued agricultural work. Pulmonary auscultation revealed isolated fine crackles and “squeaks.” Chest radiography demonstrated diffuse, fine nodular opacities bilaterally, leading the treating pulmonologist to suspect HP (initial diagnosis in 2020) as an occupational disease and report the case to the insurance provider.

A chest CT performed in 2019 was inconclusive, showing interstitial changes with subpleural reticulations and a mild craniocaudal gradient, without honeycombing, ground‐glass opacities, or mosaic attenuation. A usual interstitial pneumonia (UIP) pattern was posited by radiologists. The diagnostic criteria for HP, specifically farmer's lung in this case, were largely fulfilled, as noted by the treating physicians at that time. As symptoms exacerbated in 2020, oral prednisolone therapy (initially 40 mg daily, tapering over several weeks) was initiated.

Retrosternal pain developed but subsided after corticosteroid tapering from 40 to 7.5 mg daily following a diagnosis of osteoporosis. Pulmonary auscultation continued to reveal bilateral crackles. Spirometry and body plethysmography showed TLC and forced vital capacity (FVC) both at 47% predicted (TLC 58.6% of LLN), with a normal FEV₁/FVC ratio, indicating moderate restriction without obstruction. Chest imaging demonstrated progression of reticulonodular changes despite glucocorticoid therapy, prompting a switch to antifibrotic therapy with pirfenidone (267 mg initial, 801 mg maintenance dose, each three times daily).

Subsequent CT imaging demonstrated extensive, confluent ground‐glass and honeycomb‐like opacities without pulmonary embolism in 2021. Pirfenidone was replaced with nintedanib (150 mg twice daily), which caused persistent diarrhea even after dose reduction to 100 mg twice daily. Specific IgG testing against *Aspergillus fumigatus*, *Alternaria alternata*, timothy grass, mugwort, ribwort, birch, cat dander, and storage mites was negative. After climbing two flights of stairs, pO₂ dropped from 64.2 to 49.3 mmHg. Diffusion capacity (DLCO) was 39% predicted (52.4% of LLN), transfer coefficient (KCO) 85% predicted. Echocardiography revealed mild right atrial and ventricular dilation with preserved right ventricular systolic function.

Given the progression of restrictive impairment, hypoxemia requiring long‐term oxygen therapy, right‐heart strain, and fibrotic progression despite antifibrotic treatment, bilateral lung transplantation was performed at a university hospital in Germany in 2023 without surgical complications.

### 4.3 Clinical Course After Bilateral Lung Transplantation

Postoperatively, CMV reactivation occurred and was treated with valganciclovir. Bilateral pleural effusions required repeated drainage. Following 4 weeks of pulmonary rehabilitation, lung function and oxygenation improved, though mild restrictive dysfunction persisted.

### 4.4 Occupational Medical Assessment

In 2024, the patient was reassessed in our outpatient clinic. Histologic examination of explanted lung tissue confirmed findings compatible with HP.

Specific IgG antibodies were tested against thermophilic actinomycetes (*Micropolyspora faeni* and *Saccharopolyspora rectivirgula*, *Thermoactinomyces vulgaris*, *Thermoactinomyces sacchari*), fungi (*Aspergillus fumigatus*, *Aspergillus niger*, *Alternaria alternata*, *Cladosporium herbarum*), and other organisms, including *Candida albicans* and barley. All results were negative.

Serologic testing for HP‐associated antibodies (double immunodiffusion, ELISA, radioimmunoassays, immunoblot) can demonstrate specific IgG, but these are of limited diagnostic specificity, as over 20% of exposed but healthy individuals show positive results, and HP may occur without detectable antibodies [22]. The recent S2k guideline on HP [16] also highlights significant cross‐reactivity and nonspecific test reactions. Antigen avoidance typically results in a decline in antibody titers within 6–12 months, but complete normalization is seen in only about half of patients; systemic corticosteroids can lower total IgG levels. Given decades of documented occupational exposure, HP was again recommended for recognition as an occupational disease.

Spirometry and body plethysmography after transplantation revealed mild restriction (TLC 69% predicted, FVC 67% predicted; TLC 86.1% of LLN) without obstruction. In the 6‐min walk test, the patient achieved 73.3% of the target distance. Gas exchange and diffusion capacity (82% predicted; 109.6% of LLN) were within normal limits. Concentration remained mildly reduced, but the patient was able to climb stairs at a moderate pace without breaks. Oxygen therapy was discontinued post‐transplant due to normoxemia.

Following successful pulmonary rehabilitation, agricultural work was resumed under conditions of minimal antigen exposure (maximally achievable reduction of exposure to all agricultural antigens) and physical strain. HP was officially recognized as an occupational disease.

The primary goal in HP management is definitive antigen avoidance. At the patient's explicit request, he returned to agricultural work, as finding new employment appeared unrealistic given his age of over 60 years. Patient 3 received support from the statutory accident insurance provider, which included agricultural assistance (a helper paid for by the insurance, particularly in cases of increased risk of antigen exposure or physically demanding work) and a blower‐assisted respiratory helmet. The patient was advised to use the helmet consistently. Dust extraction systems have not yet been installed, and exposure measurements have not been conducted. Following bilateral lung transplantation, his respiratory symptoms and lung function have remained relatively stable.

## 5 Case Report 4

### 5.1 Occupational History

Approximately 40 years ago, Patient 4 (male, 40 years at initial diagnosis, 55 years at occupational assessment in our clinic, height 176 cm, weight 96 kg) completed middle school in 1984, followed by 5 years at agricultural school (1984–1989). He subsequently worked in purchasing and sales for a hardware store for 5 years (1989‐1994) and later in the administration of a fertilizer manufacturer for 6 months (1995).

He then became deputy head of the agricultural sector for 2 years (1995–1997) and head of the fertilizer and pesticides department at a hardware store for 4 years (1997–2001). Over the next 6 years (2001–2007), he worked as a field sales representative for a seed breeding company and, for the past 15 years (2007–2022), has been employed in field sales of plastic products.

In parallel, he worked full‐time on his parents' farm for 6 years (1993–1999) and, for the past 25 years (2000–2025), has continued part‐time agricultural work (approximately 15%), with subcontractors handling most of the heavy labor. The farm cultivates grain, wheat, and hops; hops are dried approximately 100 m from his residence. No visible mold growth was reported in his home.

### 5.2 Clinical Course Until Bilateral Lung Transplantation

In 1998, the patient initially developed dyspnea, wheezing, and coughing, especially early‐morning coughing attacks, which resulted in a misdiagnosis of bronchial asthma and poor treatment response. After changing pulmonologists, bronchoalveolar lavage revealed 58% lymphocytosis and a CD4/CD8 ratio of 0.4 in 2008. CT imaging showed diffuse bilateral opacities, while serum IgG antibodies to mold were highly elevated. Pulmonary function tests demonstrated moderate restriction with severely reduced diffusion capacity.

Consequently, HP was diagnosed in 2008, and the treating pulmonologist reported suspected occupational disease to the statutory accident insurance 1 month later. Recognition was initially denied because the patient's agricultural work was uninsured. Legal proceedings followed, and the court ultimately ruled that his HP should be recognized as an occupational disease, confirming that the patient's 6 years of occupational seed handling had led to significant antigen exposure (mold, thermophilic actinomycetes). Activities included decanting, mixing, and cleaning seed containers, some of which were contaminated by moisture, insects, or molds. The court deemed private agricultural exposure negligible.

**Figure 2:**
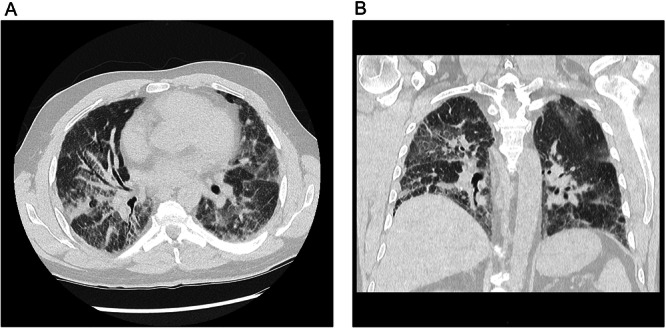
Chest computed tomography (CT) of Patient 4 (2024, before bilateral lung transplantation) in axial (A, mid‐to‐basal) and coronal (B) planes, lung window. Findings: Ground‐glass–like infiltrates, micronodules, diffuse pulmonary fibrosis, and cardiomegaly.

CT imaging revealed ground‐glass infiltrates, micronodules, diffuse fibrosis, and cardiomegaly in 2024 (Figure 2). Dyspnea, severe restriction, and severely reduced diffusion capacity progressed despite long‐term therapy with prednisolone, azathioprine, and nintedanib. Severe hypoxemia required oxygen therapy. The patient was listed for lung transplantation in 2023, and bilateral transplantation was performed in 2024 at a university hospital in Germany without perioperative complications.

### 5.3 Clinical Course After Bilateral Lung Transplantation

During a routine check‐up in 2025, the patient reported a decline in physical performance. CT imaging revealed multiple segmental pulmonary emboli in the right lower lobe without right‐heart strain. Therapeutic anticoagulation with low‐molecular‐weight heparin was initiated and continues. No bleeding has occurred. Tacrolimus trough levels fluctuated, and type 2 diabetes mellitus (HbA1c 7.8%) was newly diagnosed, prompting treatment with metformin and empagliflozin alongside continuation of oral prednisolone (10 mg daily).

### 5.4 Occupational Medical Assessment

At the last assessment (2023), the patient reported a substantial decline in exercise tolerance, with difficulty climbing stairs and a walking distance of only 250 m on level ground with supplemental oxygen.

Spirometry and body plethysmography showed moderate restriction (TLC 40% predicted, FVC 55% predicted; TLC 49.6% of LLN) without obstruction, and severely reduced diffusion capacity (37% predicted; 48.9% of LLN) with normal transfer coefficient. Resting hypoxemia (pO₂ 52.3 mmHg) declined further after minimal exertion (44.2 mmHg after 120 m walk, 16.9% of the target distance).

Given objective deterioration compared to prior evaluations, increased compensation, pulmonary rehabilitation, and follow‐up reassessment were recommended. Lung transplantation was subsequently performed in 2024, and recent data (2025) showed improved lung function (TLC 72% predicted, FVC 73% predicted, no obstruction; TLC 89.5% of LLN) and diffusion capacity (64% predicted; 84.8% of LLN). Compensation adjustments may be possible as administrative work in agriculture has resumed, supported by hired labor for physically demanding tasks. Normoxemia without supplemental oxygen was confirmed (pO₂ 71.1 mmHg), and HbA1c improved to 6.5%.

## 6 Discussion

A detailed occupational history is indispensable in patients with HP. The present case series demonstrates that clinically relevant exposures can occur in unexpected occupational settings. For instance, Patient 1 encountered avian antigens while working at a gardening center, and Patient 4 experienced substantial mold exposure as a seed dealer—occupations not typically recognized as high‐risk for HP. Similarly, craftsmen may be exposed to molds without such exposure being viewed as a classic occupational hazard. Despite this, detailed occupational histories are still often omitted from routine clinical evaluations, resulting in missed opportunities for early detection and prevention.

In Patient 2, workplace mold exposure was extensive. Although work breaks are generally not covered under statutory accident insurance in Germany, the affected basement was also used as a lounge and sleeping area during on‐call duties—justifying classification as occupational exposure. Because of the ubiquitous nature of mold, precise identification of the causal species is rarely feasible, and a direct causal relationship between exposure and disease is often difficult to prove. Nonetheless, indoor mold contamination is unacceptable for hygienic and preventive reasons and must be professionally remediated irrespective of symptomatology [23]. In this case, the extent of workplace exposure clearly exceeded private exposure, supporting recognition of HP as an occupational disease following independent occupational medical evaluation.

From an occupational medicine perspective, distinguishing legally‐recognized occupational diseases from other work‐related or non‐work‐related conditions is essential. For Patient 1, sequelae of HP must be differentiated from COPD, unless COPD arose as a secondary consequence of HP—in which case it should be considered part of the occupational disease and therefore compensable. Persistent restrictive defects and impaired diffusion capacity following bilateral lung transplantation can be explained by postoperative pleural effusions, adhesions, scarring, or organizing effusions. Moreover, pre‐existing donor lung injury cannot be excluded.

With respect to immunological testing, elevated antigen‐specific IgG levels indicate exposure rather than disease. Asymptomatic individuals may show elevated titers, while severely affected HP patients may present with normal IgG levels—particularly after a prolonged time since last exposure or during corticosteroid therapy. The recent S2k guideline for HP [16] underscores methodological limitations, variability of IgG subclass responses (especially IgG2), and the lack of standardized cut‐off values across assays and antigens. Another issue is that in some cases, there may not be commercially available tests for the inciting antigen. A German multicenter study involving 121 non‐HP subjects likewise demonstrated wide variability in specific IgG titers to 32 antigens, with the highest levels against fungal antigens such as *Aspergillus fumigatus* and *Botrytis cinerea*, largely independent of age, sex, or smoking status [24].

Comorbidities and secondary complications can further aggravate disease burden. In Patient 2, severe COVID‐19–related complications occurred after transplantation during immunosuppressive therapy. In Patient 4, corticosteroid‐induced diabetes represents a secondary consequence of occupational HP that also warrants compensation. These examples highlight the importance of comprehensive follow‐up and multidisciplinary management. Early recognition and timely reporting of occupational disease are pivotal to reducing both medical and socioeconomic consequences.

Diagnostic latency was substantial across all cases: 4 years in Patient 1, 19 years in Patient 2, 11 years in Patient 3, and 10 years in Patient 4. Such delays contributed to advanced disease necessitating bilateral lung transplantation, subsequent complications, and major impairments in quality of life. Earlier diagnosis and prompt antigen avoidance could likely have prevented these outcomes. Because HP symptoms often manifest 4–12 h after exposure—as a delayed, mixed type III/IV hypersensitivity reaction—occupational causation may not be immediately evident. Therefore, comprehensive clinical, occupational, and immunological evaluation is warranted even when initial cardiopulmonary findings appear normal.

Despite severe disease progression, vocational reintegration after transplantation is achievable. A single‐center study on lung transplantation in cystic fibrosis patients reported high employment rates within 1–10 years post‐transplantation [25]. Similarly, in our series, three of four patients successfully resumed employment, emphasizing the value of structured vocational rehabilitation alongside medical management.

Timely reporting of suspected occupational HP is of particular importance. While Patient 2 reported suspicion himself one month after diagnosis, Patient 1's case was reported only after a 4‐year delay. Early reporting facilitates financial support, accelerates medical intervention, and prevents severe complications such as respiratory failure, long‐term oxygen dependence, transplantation, immunosuppressive side effects, permanent incapacity, and premature death [16].

Occupational causes of HP must also be considered beyond traditional settings and antigens. Notably, increased incidence has been observed in workplaces with exposure to water‐based fluids and uncommon microbial contaminants, including nontuberculous mycobacteria and fungi [26]. Current guidelines should be adhered to [16].

## 7 Findings and Conclusions

- Approximately 20% of HP cases are occupationally related [13, 14].
- Delayed diagnosis and late initiation of preventive measures remain common and are associated with poor outcomes.
- Early and comprehensive pneumological evaluation, detailed occupational history, and prompt involvement of occupational medicine specialists are essential to prevent irreversible pulmonary damage and socioeconomic burden.
- Immediate antigen avoidance and early reporting of suspected occupational HP are critical to reducing morbidity, improving prognosis, and facilitating timely preventive action.

## Summary/Practical Implications

In summary, this case series underscores that hypersensitivity pneumonitis remains an underrecognized but potentially preventable occupational disease. Even in occupations not classically considered high‐risk, unrecognized antigen exposure may lead to irreversible pulmonary damage and, as illustrated here, the need for lung transplantation. Timely diagnosis requires systematic integration of occupational history into respiratory assessment, interdisciplinary collaboration between pulmonologists and occupational physicians, and early reporting to statutory insurance systems. Effective prevention and recognition not only mitigate medical and socioeconomic consequences but also enable successful vocational reintegration and sustained quality of life following advanced disease management.

## Author Contributions

**Ludwig Frei‐Stuber:** patient correspondence, data collection, manuscript writing and revision, preparation of the illustrations, patient assessment, correspondence with the editorial office. **Judith Mohren:** professional advice, patient assessment. **Ester Mau:** professional advice, patient assessment. **Bernhard Werner:** professional advice, patient assessment. **Rudolf A. Hatz:** professional advice, patient treatment. **Jürgen Barton:** professional advice, patient treatment. **Dennis Nowak:** patient correspondence, professional advice, manuscript revision, patient assessment.

## Funding

The authors received no specific funding for this work.

## Disclosure

The authors accept full responsibility for the content of this publication.

## Ethics Statement

All patients provided written informed consent for anonymous publication of their medical data. Ethics committee approval was not required for this retrospective case series.

## Conflicts of Interest

Jürgen Barton, MD, has received consulting fees from AstraZeneca and Takeda, and honoraria for lectures from AstraZeneca and Vertex. Dennis Nowak, MD, PhD; Bernhard Werner, MD; Ester Mau, MD; Judith Mohren, MPH; and Ludwig Frei‐Stuber, MD, have received fees for expert reports and lectures from statutory accident insurance providers and courts.

## References

[1] U. Costabel , F. Bonella , and J. Guzman , “Chronic Hypersensitivity Pneumonitis,” Clinics in Chest Medicine 33 (2012): 151–163. 22365252 10.1016/j.ccm.2011.12.004

[2] C. Pereira , A. Gimenez , L. Kuranishi , and K. Storrer , “Chronic Hypersensitivity Pneumonitis,” Journal of Asthma and Allergy 9 (2016): 171–181. 27703382 10.2147/JAA.S81540 PMC5036552

[3] G. Raghu , M. Remy‐Jardin , C. J. Ryerson , et al., “Diagnosis of Hypersensitivity Pneumonitis in Adults. An Official ATS/JRS/ALAT Clinical Practice Guideline,” American Journal of Respiratory and Critical Care Medicine 202 (2020): e36–e69. 32706311 10.1164/rccm.202005-2032ST PMC7397797

[4] E. R. Fernández Pérez , J. J. Swigris , A. V. Forssén , et al., “Identifying an Inciting Antigen Is Associated With Improved Survival in Patients With Chronic Hypersensitivity Pneumonitis,” Chest 144 (2013): 1644–1651. 23828161 10.1378/chest.12-2685 PMC4694094

[5] H. Barnes , J. Lu , I. Glaspole , H. R. Collard , and K. A. Johannson , “Exposures and Associations With Clinical Phenotypes in Hypersensitivity Pneumonitis: A Scoping Review,” Respiratory Medicine 184 (2021): 106444. 33991845 10.1016/j.rmed.2021.106444

[6] E. R. Fernández Pérez , A. M. Kong , K. Raimundo , T. L. Koelsch , R. Kulkarni , and A. L. Cole , “Epidemiology of Hypersensitivity Pneumonitis Among an Insured Population in the United States: A Claims‐Based Cohort Analysis,” Annals of the American Thoracic Society 15 (2018): 460–469. 29236517 10.1513/AnnalsATS.201704-288OC

[7] M. D. Mot , D. C. Olar , P. A. Vulciu , et al., “Fibrotic Hypersensitivity Pneumonitis: A Diagnostic Challenge Leading to Lung Transplantation,” Diagnostics 15, no. 10 (2025): 1267. 40428260 10.3390/diagnostics15101267 PMC12110211

[8] G. Rea , M. Bocchino , R. Lieto , et al., “The Unveiled Triad: Clinical, Radiological and Pathological Insights into Hypersensitivity Pneumonitis,” Journal of Clinical Medicine 13, no. 3 (2024): 797. 38337490 10.3390/jcm13030797 PMC10856167

[9] F. Drakopanagiotakis and P. Markart , “Interstitielle Lungenerkrankungen: Epidemiologische Herausforderungen, Register Und Biobanken,” Kompass Pneumologie 6, no. 2 (2018): 70–75.

[10] M. Kreuter , F. J. F. Herth , M. Wacker , et al., “Exploring Clinical and Epidemiological Characteristics of Interstitial Lung Diseases: Rationale, Aims, and Design of a Nationwide Prospective Registry—The Exciting‐ILD Registry,” BioMed Research International 2015 (2015): 123876. 26640781 10.1155/2015/123876 PMC4657073

[11] M. Nosotti , M. Leiva‐Juarez , F. D'Ovidio , et al., “Survival After Lung Transplantation for Chronic Hypersensitivity Pneumonitis: Results From a Large International Cohort Study,” Transplant International 35 (2022): 10450. 35431638 10.3389/ti.2022.10450 PMC9008138

[12] E. Cano‐Jiménez , A. Villar Gómez , E. Velez Segovia , et al., “Prognostic Factors of Progressive Fibrotic Hypersensitivity Pneumonitis: A Large, Retrospective, Multicentre, Observational Cohort Study,” ERJ Open Research 10, no. 1 (2024): 00405‐2023. 38410707 10.1183/23120541.00405-2023 PMC10895428

[13] M. Kreuter , J. Behr , F. Bonella , et al., “S1‐Leitlinie Interdisziplinäre Diagnostik Interstitieller Lungenerkrankungen Im Erwachsenenalter,” Pneumologie 77 (2023): 269–302. 36977470 10.1055/a-2017-8971

[14] P. D. Blanc , I. Annesi‐Maesano , J. R. Balmes , et al., “The Occupational Burden of Nonmalignant Respiratory Diseases: An Official American Thoracic Society and European Respiratory Society Statement,” American Journal of Respiratory and Critical Care Medicine 199, no. 11 (June 2019): 1312–1334, 10.1164/rccm201904-0717ST. 31149852 PMC6543721

[15] S. Quirce , O. Vandenplas , P. Campo , et al., “Occupational Hypersensitivity Pneumonitis: An EAACI Position Paper,” Allergy 71 (2016): 765–779. 26913451 10.1111/all.12866

[16] D. Koschel , J. Behr , M. Berger , et al., “Diagnostik Und Therapie Der Exogen‐Allergischen Alveolitis: S2k‐Leitlinie Der Deutschen Gesellschaft Für Pneumologie Und Beatmungsmedizin E. V. Und Der Deutschen Gesellschaft Für Allergologie Und Klinische Immunologie E. V,” Pneumologie 78, no. 12 (2024): 963–1002. 39227017 10.1055/a-2369-8458

[17] L. Frei‐Stuber , H. Drexler , A. Heutelbeck , and D. Nowak , “Obstruktive Atemwegserkrankungen Im Beruf: Wie Gelingt Eine Frühe Diagnostik Zur Vermeidung Chronischer Verläufe?,” Pneumologie 77, no. 6 (2023): 350–356. 37068510 10.1055/a-2055-0806

[18] L. Frei‐Stuber , A. Wächter , T. Benthaus , U. Ochmann , and D. Nowak , “Prävention Eines Progresses Des Allergischen Asthma Bronchiale Als Berufskrankheit,” Pneumologie 78, no. 4 (2024): 269–275. 37857319 10.1055/a-2185-0896

[19] G. M. Klaut , S. Karrasch , S. Kutzora , D. Nowak , and C. Quartucci , “The Impact of Years of Training and Possible Technical, Procedural, and Individual Risk Factors for the Development of Atopic Symptoms Among Bakery and Confectionery Trainees,” International Archives of Occupational and Environmental Health 97, no. 7 (2024): 721–731. 38951216 10.1007/s00420-024-02079-7

[20] G. M. Klaut , L. Frei‐Stuber , S. Karrasch , et al., “Longitudinal Study of Fractional Exhaled Nitric Oxide (FeNO) and Spirometric Indices in Trainee Bakers and Confectioners,” Clinical and Experimental Allergy 55, no. 10 (2025): 945–948. 40364670 10.1111/cea.70076 PMC12515534

[21] P. H. Quanjer , G. L. Hall , S. Stanojevic , T. J. Cole , and J. Stocks , “Age‐ and Height‐Based Prediction Bias in Spirometry Reference Equations,” European Respiratory Journal 40, no. 1 (2012): 190–197. 22183491 10.1183/09031936.00161011

[22] Bundesministerium für Arbeit und Sozialordnung . Merkblatt zur BK Nr. 4201: Exogen‐allergische Alveolitis. In: Ärztlicher Sachverständigenbeirat Berufskrankheiten, ed. Vol 11. Berlin: Bundesarbeitsblatt; 1989:63.

[23] Umweltbundesamt . Leitfaden zur Vorbeugung, Erfassung und Sanierung von Schimmelbefall in Gebäuden. In: Innenraumlufthygiene‐Kommission des Umweltbundesamtes, ed. Dessau‐Roßlau2017 (Update April 2024).

[24] M. Raulf , M. Joest , I. Sander , et al., “Update of Reference Values for IgG Antibodies Against Typical Antigens of Hypersensitivity Pneumonitis,” Allergo Journal International 28, no. 6 (2019): 192–203.

[25] T. Radtke , A. Königs , X. Chen , J. Braun , H. Dressel , and C. Benden , “Predictors of Long‐Term Employment Among Patients With Cystic Fibrosis Undergoing Lung Transplantation,” Swiss Medical Weekly 150 (2020): w20286. 32667678 10.4414/smw.2020.20286

[26] K. Kreiss and J. Cox‐Ganser , “Metalworking Fluid‐Associated Hypersensitivity Pneumonitis: A Workshop Summary,” American Journal of Industrial Medicine 32, no. 4 (1997): 423–432. 9258399 10.1002/(sici)1097-0274(199710)32:4<423::aid-ajim16>3.0.co;2-5

